# The impact of a microsavings intervention on reducing violence against women engaged in sex work: a randomized controlled study

**DOI:** 10.1186/s12914-016-0101-3

**Published:** 2016-10-28

**Authors:** Laura Cordisco Tsai, Catherine E. Carlson, Toivgoo Aira, Andrea Norcini Pala, Marion Riedel, Susan S. Witte

**Affiliations:** 1 George Mason University College of Health and Human Services, MSN 1F8, Fairfax, VA 22030 USA; 2 New York State Psychiatric Institute, Columbia University, 1051 Riverside Drive, New York, NY 10032 USA; 3 Wellspring NGO, Zorig Foundation Building, Peace Avenue 9A, Sukhbaatar District, Ulaanbaatar, Mongolia; 4 Columbia University, HIV Center for Clinical Behavioral Studies at the New York State Psychiatric Institute and Columbia University, New York, NY 10032 USA; 5 Columbia University School of Social Work, 1255 Amsterdam Avenue, New York, NY 10027 USA

**Keywords:** Gender-based violence, Microfinance, Economic empowerment, Randomized controlled trial, Central Asia

## Abstract

**Background:**

Women who engage in sex work are at risk for experiencing violence from numerous perpetrators, including paying partners. Empirical evidence has shown mixed results regarding the impact of participation in microfinance interventions on women’s experiences of violence, with some studies demonstrating reductions in intimate partner violence (IPV) and others showing heightened risk for IPV. The current study reports on the impact of participation in a microsavings intervention on experiences of paying partner violence among women engaged in sex work in Mongolia.

**Methods:**

Between 2011 and 2013, we conducted a two-arm, non-blinded randomized controlled trial (RCT) comparing an HIV/STI risk reduction intervention (HIVSRR) (control condition) to a combined microsavings and HIVSRR intervention (treatment condition). Eligible women (aged 18 or older, reported having engaged in unprotected sex with paying partner in past 90 days, expressed interest in microsavings intervention) were invited to participate. One hundred seven were randomized, including 50 in the control and 57 in the treatment condition. Participants completed assessments at baseline, immediate post-test following HIVSRR, and at 3-months and 6-months after completion of the treatment group intervention. Outcomes for the current study include any violence (physical and/or sexual), sexual violence, and physical violence from paying partners in the past 90 days.

**Results:**

An intention-to-treat approach was utilized. Linear growth models revealed significant reductions over time in both conditions for any violence (β = −0.867, *p* < 0.001), physical violence (β = −0.0923, *p* < 0.001), and sexual violence (β = −1.639, *p* = 0.001) from paying partners. No significant differences between groups were found for any violence (β = 0.118, *p* = 0.389), physical violence (β = 0.091, *p* = 0.792), or sexual violence (β = 0.379, *p* = 0.114) from paying partners.

**Conclusions:**

Microsavings participation did not significantly impact women’s risk for paying partner violence. Qualitative research is recommended to understand the cause for reductions in paying partner violence in both study conditions.

**Trial registration:**

Evaluating a Microfinance Intervention for High Risk Women in Mongolia; NCT01861431; May 20, 2013.

## Background

Women who engage in sex work are vulnerable to violence from multiple perpetrators, including intimate partners, police, managers, and paying partners/customers [1–5]. Various studies have found 45 % to 76 % of women who engage in sex work experience paying partner violence in their lifetime [3, 6, 7]. Women who engage in street-based sex work, women who are new to sex work, and younger women are at particularly high risk for paying partner violence [5, 8]. The stigmatized nature and illegal status of sex work in most countries inhibit women’s ability to seek protection from formal or informal sources [6].

Recent studies have found high prevalence of violence against women engaged in sex work in Mongolia. Carlson and colleagues (2012) found that 56 % of women engaged in street-based sex work in Ulaanbaatar, Mongolia reported experiencing physical violence from paying partners and 33 % reported experiencing sexual violence from paying partners in the past 90 days [9]. In contrast, a study pertaining to violence against women in the general population of Mongolia found that about 18 % of women reported experiencing physical violence and 10 % reported experiencing sexual violence in the past 6 months [10]. Paying partner violence holds many detrimental consequences for women. Exposure to paying partner violence places women at higher risk for physical and behavioral health issues, including elevated HIV/STI exposure, alcohol and substance use/abuse, unwanted pregnancy, abortion, and physical injuries, among others [3, 5, 6, 8, 9, 11]. A recent study in Mongolia found that recent sexual violence from paying partners was positively associated with having engaged in recent unprotected vaginal or anal sex with paying partners [12]. Additionally, paying partner violence impacts the mental health of women, leading to anxiety, depressive symptoms, suicidal thoughts and attempts, and post-traumatic stress disorder [5, 6, 11]. The complex, intersectional issues experienced by women engaged in sex work make developing and targeting health or mental health interventions for this population particularly challenging. Increasingly, health advocates are calling for structural interventions that may address multiple issues, including poverty alleviation strategies such as microfinance. Prior evaluations of microfinance interventions with women engaged in sex work have found that microfinance participation led to significant reductions in sexual risk behavior and decreased engagement in sex work [13–16]. However, care must be taken when developing these public health interventions to not inadvertently increase risk for violence [17].

Empirical evidence has shown varying results regarding the relationship between women’s experiences of violence and participation in microfinance interventions. Some studies have found women’s participation in microfinance interventions to be protective from intimate partner violence (IPV) [18, 19], while others have shown that their participation in microfinance programs can increase their risk for IPV [20–22]. Other studies report mixed results regarding the impact of participation in microfinance interventions on women’s experiences of IPV [23–25]. There are, however, numerous gaps in the existent literature when examining the experiences of women engaged in sex work specifically. While the current literature speaks to the impact of microfinance on women’s experiences of IPV, to the authors’ knowledge, no studies have addressed the impact of microfinance participation on experiences of paying partner violence among women engaged in sex work specifically. Further, the vast majority of the aforementioned studies pertain to microcredit/microenterprise interventions. Significantly less attention has been paid to the impact of microsavings interventions on women’s experiences of violence, even though savings-led approaches to microfinance have been increasing in popularity and recognition in recent years [26]. The purpose of the current study was to examine the efficacy of a combined HIV/STI sexual risk reduction (HIVSRR) plus microsavings condition and a HIVSRR alone condition at reducing recent paying partner violence against women engaged in street-based sex work in Mongolia.

## Methods

**Fig. 1:**
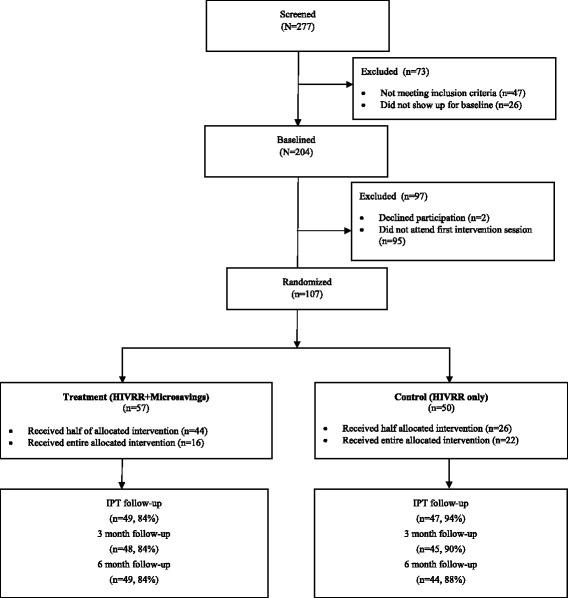
Consolidated Standards of Reporting Trials (CONSORT) Diagram of Participant Enrollment

Data from this paper come from a randomized trial that was implemented from 2011 to 2013 in Ulaanbaatar, Mongolia with 107 women engaged in street-based sex work [27]. The current study took place as part of a parent study testing the efficacy of adding a microfinance component following an HIV risk reduction intervention on women’s sexual risk and economic dependence upon sex work. Women (*n* = 107) were recruited and randomized to either (a) a relationship-based HIV sexual risk reduction intervention (HIVSRR), or (b) the same sexual risk reduction intervention plus a microsavings intervention, as shown in Fig. 1. The parent study found the combined HIVSRR and microsavings intervention to be effective in reducing sexual risk [27] and reducing women’s economic dependence upon sex work [28]. Although the microsavings intervention was not originally targeted to reduce violence, the study team hypothesized that a reduction in paying partner violence would be observed in the treatment group since the microsavings intervention appeared to be effective in helping women transfer some of their income from sex work to other sources [28]. We hypothesized that reduced engagement in sex work may be accompanied by reduced exposure to paying partner violence.

We calculated power to detect significant differences using repeated measure ANOVA procedures with 1 pre-intervention (i.e., “baseline”) and 3 post-intervention assessments. In the absence of preliminary estimates, power was calculated (G*POWER software) as a function of effect size for a range of within subject correlation (ρ); the range of ρ values used in the analyses span those observed in the investigative team’s previous randomized trial among women engaged in sex work, which used an identical assessment schedule and sexual risk outcome measures [29]. Results indicate that with 100 participants or 50 subjects per condition, 80 % power is achieved for effect sizes f^2^ between .06 and .08.

We used a targeted sampling strategy in Ulaanbaatar and the peri-urban ger district^1^ to enhance generalizability of findings to women working in sex work in Ulaanbaatar [30]. The study team first developed a list of street-based sex work venues identified by participants in prior studies and key stakeholders involved in prior studies, including leaders from NGOs serving women engaged in sex work [29]. The study team conducted multiple observations per site, recording the number of women seen soliciting at each site. These observations were then used to develop a proportional sampling strategy, as recommended by Peterson and colleagues [30]. Further details regarding the proportional sampling approach utilized in the study are presented elsewhere [27, 28].

During recruitment, trained outreach workers (including women engaging in sex work) approached women at sex work sites, provided information on the study, and completed a brief screening interview with interested women. Women were eligible if they were: 1) at least 18 years of age; 2) reported having engaged in sexual intercourse in the past 90 days in exchange for money, alcohol or other goods; 3) reported having engaged in unprotected sexual intercourse in the past 90 days with a paying sexual partner; and 4) reported interest in learning about and developing additional income sources. After screening, eligible women completed informed consent procedures and a baseline assessment administered by trained interviewers. All women who completed the baseline assessment (*n* = 204) were invited to participate in study sessions. A total of 107 women attended the first study session and were randomized to one of the two study conditions, reflecting an uptake of 52 %. All study protocols were reviewed and approved by the Institutional Review Boards at the National University of Mongolia in Ulaanbaatar and Columbia University. Signed consent was waived in light of the stigma associated with women’s engagement in sex work. Verbal consent was obtained instead and women were given a copy of the informed consent form.

### Intervention

The control condition, the HIVSRR intervention, consisted of a 4-session intervention successfully adapted and tested among women engaged in sex work in Mongolia in a previous study [29]. The four HIVSSR sessions focused on building knowledge and skills on how to protect themselves and safely engage paying partners in sexual risk reduction, such as identifying safety risks, negotiating safer sex, and avoiding unsafe situations. For example, women were shown a video depicting specific ways to protect oneself from violence, such as telling a friend where you are going with a client, avoiding completely private spaces, not getting into a car with a client, and not getting into a car with more than one client.

The treatment condition, which was based on social cognitive and asset theories, consisted of the aforementioned 4-session HIVSRR intervention plus a microsavings intervention, which was successfully piloted with promising results prior to study implementation [31]. Fundamental to Social Cognitive Theory (SCT) is the concept of self-efficacy, which impacts whether people consider changing their behavior, the level of effort they invest in behavioral change, and long-term maintenance of behavior change [32, 33]. We designed the treatment intervention around building self-efficacy for financial literacy, savings, and vocational development. For example, in the training component of the treatment condition, we included practice role-plays and knowledge modeling to integrate key financial skills, such as budgeting, financial negotiation, and building a savings plan. Moreover, the study team adapted the training by integrating goal-setting activities at the end of each session to generalize lessons. The intervention was also informed by asset theory, which posits that assets influence not only a person’s economic situation, but also their behaviors, attitudes, and perception of the future. Therefore, the accumulation of assets can result in psychological, social, and behavioral benefits in addition to heightened economic stability and improvement of household economic circumstances [34, 35].

Many microfinance interventions for women working in sex work have adopted a microcredit model, which refers to the provision of small loans for small business development. Women facing pressure to repay loans may have no other option but to use income from non microfinance-related sources to pay their debts [36], which may increase risk behavior for women already engaging in high-risk transactional sex. In the current study, we utilized a microsavings approach. Microsavings refers to a branch of microfinance in which small deposit accounts or collection mechanisms are offered to low-income individuals/households in order to support participants in building savings for future use. Without a loan component, microsavings is less financially risky than microcredit as it fosters asset development without burdening clients with high interest rates or over-reliance upon debt [37].

The microsavings intervention consisted of a matched savings program–an evidence-based intervention that promotes asset building–and financial training [38]. As a part of the matched savings component, each participant had the opportunity to open a savings account at a partner financial institution that had been pre-screened prior to study implementation for accessibility to marginalized populations. Money that the women deposited in their savings accounts was matched on a 1:1 basis, up to a maximum of 60,000 Mongolian Tugriks (MNT) per month (or approximately 40 USD per month), during the 4 months of the matched savings program (or a maximum of 240,000 MNT throughout the intervention). Matched savings could be used for two purposes: 1) start-up capital for small business development or 2) job/vocational training. The matched savings process incentivized participants to save and enabled them to build assets more quickly without the risks associated with microcredit [36, 37].

The training program consisted of 12 sessions on financial literacy, 12 sessions on business development, and 10 sessions of vocational mentorship. To be eligible to access the matched savings, participants were required to attend 9 of 12 financial literacy training sessions, 9 out of 12 business development training sessions, and 6 of 10 mentorship sessions. The financial literacy training, adapted from the *Global Financial Education Program* [39], included material on bank services, savings, budgeting, debt management and financial negotiation. The business development training was adapted from the International Labour Organization (ILO)’s *Gender and Entrepreneurship Together* curriculum, and included sessions pertaining to business planning, entrepreneurship, social networking skills, and gender-based issues impacting women’s access to business opportunities [40]. The final component of the training program entailed group mentorship sessions wherein local female business owners and study team members met with participants to support participants in reaching their vocational goals.

In light of concerns mentioned in prior literature regarding the connection between microfinance participation and experiences of violence, check-ins regarding paying and intimate partner violence were integrated into training sessions [20, 22, 25]. All staff were trained to implement safety planning and provide community resources and referrals if participants disclosed experiences of violence. Session by session check-ins yielded no participant reports of concern regarding violence.

### Assessments

Participants completed computer-based assessments administered by trained interviewers at four time points: at baseline, immediate post-test (IPT) following the HIVSRR sessions, and at 3-months and 6-months after completion of the treatment group intervention. All assessments were conducted between November 2011 and July 2013. Three and 6-month assessments measured all variables from the baseline assessment. Both conditions began with the 4-session HIVSRR, and the IPT was incorporated to measure the short-term impact of the HIVSRR on sexual risk outcomes only. As a result, the IPT was dropped from the final analyses. Participants were compensated financially for their time. Women received 2000 MNT^2^ (approximately 1.54 USD) for the screening, 6000 MNT (approximately 4.62 USD) for the baseline interview and for each intervention session, 15,000 MNT (approximately 11.54 USD) for the 3-month follow-up, and 18,000 MNT (approximately 13.85 USD) for the 6-month follow-up.

Baseline socio-demographic variables reported in this paper include the women’s age, educational attainment, whether or not the woman reported having an intimate partner (such as a spouse), the number of years engaged in sex work, whether or not sex work was the woman’s main source of income, and level of harmful alcohol use. The latter was measured by using the Alcohol Use Disorders Identification Test (AUDIT), a ten-item scale that ranks a respondent’s self-reported alcohol use from 0 to 40, with 40 being the most harmful possible score [41].

Outcome variables at 3-month and 6-month follow-up included the following: 1) any experience of paying partner violence (physical and/or sexual violence) in the past 90 days, 2) experience of physical paying partner violence in the past 90 days, and 3) experience of sexual paying partner violence in the past 90 days. To examine paying partner violence, an adaptation of the Revised Conflict Tactics Scale (CTS2) was used to measure violence, assessing “lifetime” and “past 90 days” violence at the hands of paying partners [42]. Only violence experienced in the past 90 days, hereafter referred to as “recent paying partner violence,” was utilized in this analysis due to the time frame of the intervention. Since the treatment group intervention lasted longer than 90 days, any violence the women reported they had experienced in the past 90 days at 3-month and 6-month follow-up should have taken place after they completed their assigned intervention.

### Data analysis

An intention-to-treat approach was utilized. To test the hypothesis that participation in the HIVSRR plus microsavings treatment condition resulted in a greater reduction in paying partner violence than participation in the HIVSRR only control condition, we performed growth model analysis to identify a trajectory that described the change in the probability of experiencing recent paying partner violence. We conducted three separate growth model analyses–one addressing any recent paying partner violence (physical and/or sexual), a second model addressing any recent physical paying partner violence, and a third model addressing any recent sexual paying partner violence. We did not adjust for covariates, as we found no significant differences in key predictors at baseline, which included income, age, level of education, whether or not the women reported having an intimate partner, years engaged in sex work, and AUDIT scores. Since the outcome variables were binary 0/1, where zero corresponded to absence of violence and one presence of violence, the trajectory is based on the probability, ranging from 0 to 1. The difference between the two groups was evaluated by including a variable that identified the two groups within the growth model. The slope (mean change in the probability to report recent paying partner violence) and intercept (baseline probability to report recent paying partner violence) of the trajectory were both regressed on the treatment arm. A statistically significant effect of the treatment arm on the slope would confirm a statistically significant difference between the two groups on the change of valid prediction period (VPP) probability. A statistically significant effect of the treatment arm on the intercept would indicate statistically significant difference levels of violence at baseline. The growth model was tested with the Maximum likelihood with Robust Standard Error (MLR) instead of the default estimator for binary/categorical outcomes Weighted Least Square Mean and Variance (WLSMV) since the outcome variables showed a small amount of missing data. Whereas MLR addresses the missing data by treating them as missing at random (MAR), WLSMV does not [43].^3^

## Results

Among the 107 participants, 57 women participated in the microsavings treatment group and 50 participated in the control group. We found no significant difference in baseline values of recent paying partner violence, age, income, and intimate partner relationships (yes/no) between the women who were randomized and those who were not. The retention rate was 87 % for both the 3-month and 6-month follow-up assessments. Six-month attrition rates did not significantly differ by condition (*χ* ^2^ = 0.10, *p* = 0.76). We examined the association between missing data and baseline variables. We found no significant differences between complete and missing cases in regard to baseline levels of physical paying partner violence (*χ* ^2^ = 0.01, *p* = 0.93), sexual paying partner violence (*χ* ^2^ = 0.00, *p* = 1.00), relationship status (*χ* ^2^ = 2.01, *p* = 0.15), mean income (*t* = 0.45, *p* = 0.65), or mean educational level (*t* = 0.34, *p* = 0.74). Complete cases were on average significantly older than cases with missing data (*t* = 2.53, *p* < 0.05).

The average age of participants was 36, most had completed secondary school and approximately half reported having a intimate partner (see Table 1). Women reported working in sex work for an average of 6 years, and over 90 % of the women indicated that sex work was their main source of income at baseline. Almost 80 % of women reported harmful alcohol use. Differences in demographic characteristics or baseline values of risk variables did not significantly differ by condition, suggesting that randomization was successful. There were no statistically significant differences between the two groups with respect to experiences of any, physical, and sexual recent paying partner violence reported in the past 90 days at baseline.

**Table 1:** Socio-demographic characteristics of study participants at baseline

	HIVSRR control group (*n* = 50)	HIVSRR + microsavings treatment group (*n* = 57)	Total (*n* = 107)	*χ* ^*2*^ *or t*
	n (%) or *M* (*SD*)	n (%) or *M* (*SD*)	n (%) or *M* (*SD*)	
Age	37.7 (9.3)	35.7 (9.3)	36.6 (9.3)	1.12
Level of education				3.76
Primary or none	5 (10.0)	3 (5.2)	8 (7.4)	
Secondary	32 (64.0)	46 (80.7)	78 (72.8)	
More than secondary	13 (26.0)	8 (14.0)	21 (19.6)	
Has intimate partner	21 (42.0)	28 (49.1)	49 (45.8)	0.54
Years in sex work	5.7 (4.8)	6.9 (5.6)	6.4 (5.2)	−1.18
Sex work as main income source (yes)	46 (92.0)	53 (92.9)	99 (92.5)	0.04
AUDIT scores				3.65
Zone I (0–7)	10 (20.0)	12 (21.1)	22 (20.5)	
Zone II (8–15)	8 (16.0)	15 (26.3)	23 (21.4)	
Zone III (16–19)	6 (12.0)	10 (17.5)	16 (14.9)	
Zone IV (20–40)	26 (52.0)	20 (35.1)	46 (42.9)	

A clear and considerable drop in reports of recent paying partner violence was observed in both groups from baseline to 3 and 6-month follow-up. As shown in Table 2, 43.9 % of women reported having experienced any paying partner violence in the past 90 days at baseline, including 39.5 % of the total sample who reported physical violence and 25.2 % percent who reported sexual violence. Reports of recent paying partner violence declined at each subsequent time point. By 6-month follow-up, 11.8 % of women reported having experienced any paying partner violence in the past 90 days, including 10.8 % who reported physical violence and 7.5 % who reported sexual violence.

**Table 2:** Proportion of women in each condition experiencing paying partner violence across three time points

Time point	Violence experienced in the past 90 days	HIV-SRR control group	HIV-SRR + microsavings treatment group	Total	
		n (%)	n (%)	n (%)	*χ* ^*2*^
Baseline (*n* = 107)	Any violence	24 (48.0)	23 (40.4)	47 (43.9)	0.63
	Physical	21 (42.0)	21 (36.8)	42 (39.5)	0.30
	Sexual	15 (30.0)	12 (21.1)	27 (25.2)	1.13
3 month follow-up (*n* = 93)	Any violence	8 (17.8)	6 (12.5)	14 (15.1)	0.51
	Physical	5 (11.1)	6 (12.5)	11 (11.8)	0.04
	Sexual	4 (8.9)	4 (8.3)	8 (8.6)	0.01
6 month follow-up (*n* = 93)	Any violence	4 (9.1)	7 (14.3)	11 (11.8)	0.60
	Physical	4 (9.1)	6 (12.2)	10 (10.8)	0.24
	Sexual	2 (4.5)	5 (10.2)	7 (7.5)	1.07

### Any violence growth model

**Fig. 2:**
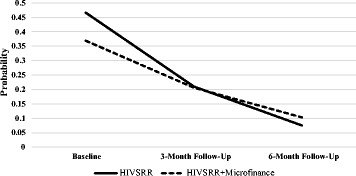
Probability of experiencing any recent paying partner violence over time by condition

The linear growth model fit the data well as confirmed by *χ* ^2^ test of fit for binary outcomes (Pearson *χ* ^2^ = 0.662, df = 2, *p* = 0.718 and Likelihood ratio *χ* ^2^ = 1.474, df = 2, *p* = 0.479). A trajectory with a slope statistically significantly different from zero was identified; that is, overall there was a significant drop in reporting any recent paying partner violence (physical and/or sexual). See Fig. 2 for a graphical representation of the trajectory. The standardized slope mean was −0.867 (*p* < 0.001), which means that over the 6 months a significant drop in the reported experience of any recent paying partner violence was observed. The slope and intercept variance was not statistically significant (*D*_*s*_ = 4.011, *p* = 0.230 and *D*_*i*_ = 40.314, *p* = 0.205), suggesting that there was not a significant difference across individuals. The effect of the treatment arm variable was statistically non-significant on both intercept (β = −0.124, *p* = 0.525) and slope (β = 0.118, *p* = 0.389), as shown in Table 3. Therefore, the two groups did not differ at baseline on experiences of any recent paying partner violence and on the change of recent paying partner violence rates over the 6 months.

**Table 3:** Linear growth models of experiencing recent paying partner violence

Growth model	Standardized slope (SE)	Effect of treatment arm on intercept (SE)	Effect of treatment arm on slope (SE)
Any violence^a^ growth model	−0.867 (0.190)***	−0.124 (0.196)	0.118 (0.137)
Physical violence growth model	−0.923 (0.181)***	−0.070 (0.167)	0.091 (0.345)
Sexual violence growth model	−1.639 (0.481)***	−0.300 (0.253)	0.379 (0.239)

***p ≤ 0.001; *SE* Standard Error

^a^ Physical and/or sexual violence

### Physical violence growth model

**Fig. 3:**
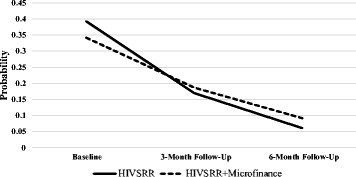
Probability of experiencing recent physical paying partner violence over time by condition

The *χ* ^2^ tests of fit for binary outcomes confirmed a good fit (Pearson *χ* ^2^ = 4.743, df = 2, *p* = 0.093 and Likelihood ratio *χ* ^2^ = 3.446, df = 2, *p* = 0.178). The standardized slope of the trajectory was −0.0923 (*p* < 0.001), demonstrating a significant reduction in reports of recent physical violence over time in both groups (as shown in Fig. 3). The variance of the slope and intercept were not statistically significant (*D*_*s*_ = 6.157, *p* = 0.342 and *D*_*i*_ = 36.124, *p* = 0.494). Therefore, no significant variability across individuals was observed. The two groups did not differ in a statistically significant manner (effect of the treatment arm on the intercept and slope was respectively β = −0.070, *p* = 0.673 and β = 0.091, *p* = 0.792), as per Table 3.

### Sexual violence growth model

**Fig. 4:**
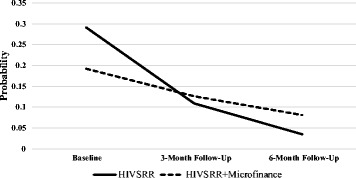
Probability of experiencing recent sexual paying partner violence over time by condition

Lastly, sexual violence outcomes were analyzed and the linear model fitted the data well (Pearson *χ* ^2^ = 0.517, df = 2, *p* = 0.772 and Likelihood ratio *χ* ^2^ = 0.602, df = 2, *p* = 0.74). The standardized slope of the trajectory was −1.639 (*p* = 0.001), showing a significant reduction in reports of recent sexual violence over time, as demonstrated in Fig. 4. No variability across participants was observed as confirmed by the slope and intercept variance (*D*_*s*_ = 0.232, *p* = 0.396 and *D*_*i*_ = 0.932, *p* = 0.566). As reflected in Table 3, the two groups did not differ in a statistically significant manner (effect of the treatment arm on the intercept and slope was respectively β = −0.300, *p* = 0.235 and β = 0.379, *p* = 0.114).

## Discussion

This study represents, to the authors’ knowledge, the first randomized trial exploring the impact of microfinance participation on experiences of violence among women engaged in sex work. While other studies have explored the impact of microfinance on women’s experiences of intimate partner violence, our study is unique in that it focuses specifically on the experiences of women engaged in sex work and their experiences of violence from paying partners. Furthermore, prior research pertaining to the impact of microfinance on women’s experiences of violence has largely examined microcredit interventions. In light of concerns regarding the risks associated with credit-based approaches to microfinance [37, 44], our study utilized a microsavings approach, allowing for asset development without the financial risks associated with acquiring debt [28]. As such, the current study contributes to the evidence base by evaluating a different model for providing financial services and exploring the implications for experiences of violence among a particularly vulnerable and marginalized group of women.

Study findings reveal significant reductions in paying partner violence against women who engage in sex work in Mongolia in both study conditions. Contrary to our hypothesis, participation in a microsavings intervention did not reduce exposure to paying partner violence. The findings from our study differ from prior studies that have found microfinance participation to be protective from violence, specifically intimate partner violence [18, 19]. However, examining findings in light of current literature is complicated by the uniqueness of this study, i.e. that it examines microsavings instead of microcredit and that we examine paying partner violence as opposed to intimate partner violence. Given that participation in this microsavings intervention was found to be effective in transferring women’s income from sex work to other sources [28], it is unclear why women’s decreased engagement in sex work was not accompanied by decreased exposure to paying partner violence. It is possible that increasing income from alternative (non-sex work) sources may not prevent women from continuing to experience violence from particularly abusive clients. Further, women who were able to increase their income from alternative sources may not have been at the highest risk for paying partner violence. As the first study of its kind, these findings raise important questions for future practice and research on the impact of microsavings on preventing violence against women engaged in sex work. Qualitative research is recommended in order to understand the experiences of paying partner violence among microsavings participants, particularly among those who reduced their sex work engagement.

Given the significant reductions in paying partner violence observed in both conditions, it is worth considering alternative explanations for change coming from outside the original model, such as environmental events or the development of social support networks. Peer networks can be an important source of emotional support for women in sex work and can help connect women with other resources in the community [45, 46]. Prior to joining this intervention, participants had minimal contact or support from social services. In an under-resourced environment in which few support services are available for this highly marginalized and stigmatized population, it is possible that the development of peer networks and support from professionals may have contributed to a reduction in exposure to violence for women in both study conditions. Further, a prior study demonstrated that the HIVSRR intervention may have been effective in reducing paying partner violence against women who engage in sex work in Mongolia [9]. While it is possible that participation in the HIVSRR portion of the intervention may have led to reductions in violence, this cannot be assessed in the current study due to the lack of an unexposed control condition.

Study limitations include the study’s relatively small sample size and self-report of assessments. Although the 90-day recall period for the outcome variables is common in violence research, it may be subject to poor participant recall. Further, the study did not involve an unexposed control group and thus we do not know what would have happened in the absence of any intervention. Economic dependence upon sex work may have served as a mediator. However, a mediation analysis was not conducted due to the small sample size. Additionally, the study was not originally powered to look for a reduction in violence.

Attendance or dose of intervention was low overall; session attendance ranged from 3 to 100 %. Average attendance was higher in the HIVSRR sessions (76 % of sessions), in comparison to the average attendance in the microfinance-related sessions (46 %). Low attendance reflects the multiple barriers experienced by women engaged in sex work in low-resourced settings and challenges related to implementation of more time-intensive interventions. Although a preliminary dose analysis revealed that attendance did not have a significant effect on the slope of the trajectories, a potential ‘dose’ effect should be more thoroughly investigated using a larger sample size.

The study only explored paying partner violence, but did not address violence from other perpetrators such as intimate partners, law enforcement, or pimps/bosses. As discussed earlier, women who engage in sex work are vulnerable to multiple forms of gender-based violence [1–3, 5]. It is possible that microsavings participation could increase women’s risk for violence at the hands of non-paying intimate partners, since a change in occupation status or level of independence may be perceived as threatening to intimate partners. Additionally, while only five study participants reported having a ‘boss’/pimp, it is plausible that the act of transitioning to alternative income sources could place women at risk of heightened violence from bosses/pimps. Future research should consider violence from numerous perpetrators from whom women in sex work may experience violence, including brokers/pimps, intimate partners, and police, in order to best target interventions and reduce risk from multiple forms of violence.

## Conclusions

The current study holds important implications for research and practice pertaining to microfinance, sexual risk reduction, and violence prevention initiatives among women engaged in sex work. The study engaged a highly vulnerable group of women in an HIVSRR and microsavings intervention without increasing reported experiences of violence from paying partners. Given prior research that has found microfinance participation to increase women’s risk for violence [20–22, 25], such demonstration should not be underrated. Ethical considerations for the potential to increase danger among groups at high risk for violence must be weighed against gains achieved in any research endeavor. Small careful steps that guide us closer to the evolution of interventions assuring the safety of such a vulnerable population offer value to the field.

## Abbreviations

AUDIT: Alcohol Use Disorders Identification Test
CTS2: Revised Conflict Tactics Scale
HIVSRR: HIV/STI risk reduction intervention
ILO: International Labour Organization
IPT: Immediate post-test
IPV: Intimate partner violence
MAR: Missing at random
MLR: Robust Standard Error
MNT: Mongolian Tugriks
RCT: Randomized controlled trial
VPP: Valid prediction period
WLSMV: Weighted Least Square Mean and Variance

## Footnotes

1 A ger is a circular residence that is constructed with wooden frames and felt walls. Gers have traditionally been used as the dwelling place of nomadic peoples in Mongolia. In urban Ulaanbaatar, residents construct gers in congregated areas called ger districts.

2 At the time of developing this intervention, the conversion rate between MNT and USD was roughly 1300 MNT per USD. The conversion rate changed throughout the project, as inflation was volatile in Mongolia during the study.

3 Model fit indexes such as RMSEA and CFI/TLI are not available when binary data are analyzed with MLR estimator.
